# Supplementary material for: Animal life in the shallow subseafloor crust at deep-sea hydrothermal vents
Source: Nat Commun. 2024 Oct 15;15:8466. doi: 10.1038/s41467-024-52631-9 (PMC11480316; doi:10.1038/s41467-024-52631-9)
Supplement: Supplementary file 2 — Description of Additional Supplementary Files [file 41467_2024_52631_MOESM2_ESM.pdf]

Description of Additional Supplementary Files

File Name: Supplementary Movie 1
Description: Lifting of lobate lava shelves at the hydrothermal vent site Fava Flow Suburbs at 9°50'N EPR at 2515 m depth in July 2023. Clip 1 dive S0552, clip 2 S0554, clip 3 S0560, clip 4 and 5 S0556, clip 6 S0557 (S0558 not shown due to lack of animals in very shallow cavity).
