# Supplementary material for: Animal life in the shallow subseafloor crust at deep-sea hydrothermal vents
Source: Nat Commun. 2024 Oct 15;15:8466. doi: 10.1038/s41467-024-52631-9 (PMC11480316; doi:10.1038/s41467-024-52631-9)
Supplement: Supplementary file 4 — Reporting Summary [file 41467_2024_52631_MOESM4_ESM.pdf]

nature portfolio

Corresponding author(s): Monika Bright
Sabine Gollner

Last updated by author(s): 8/26/2124

Reporting Summary

Nature Portfolio wishes to improve the reproducibility of the work that we publish. This form provides structure for consistency and transparency in reporting. For further information on Nature Portfolio policies, see our Editorial Policies and the Editorial Policy Checklist.

Statistics

For all statistical analyses, confirm that the following items are present in the figure legend, table legend, main text, or Methods section.

n/a	Confirmed	
[ ]	[x]	The exact sample size (*n*) for each experimental group/condition, given as a discrete number and unit of measurement
[x]	[ ]	A statement on whether measurements were taken from distinct samples or whether the same sample was measured repeatedly
[ ]	[x]	The statistical test(s) used AND whether they are one- or two-sided<br>*Only common tests should be described solely by name; describe more complex techniques in the Methods section.*
[x]	[ ]	A description of all covariates tested
[x]	[ ]	A description of any assumptions or corrections, such as tests of normality and adjustment for multiple comparisons
[ ]	[x]	A full description of the statistical parameters including central tendency (e.g. means) or other basic estimates (e.g. regression coefficient) AND variation (e.g. standard deviation) or associated estimates of uncertainty (e.g. confidence intervals)
[ ]	[x]	For null hypothesis testing, the test statistic (e.g. *F*, *t*, *r*) with confidence intervals, effect sizes, degrees of freedom and *P* value noted<br>*Give P values as exact values whenever suitable.*
[x]	[ ]	For Bayesian analysis, information on the choice of priors and Markov chain Monte Carlo settings
[x]	[ ]	For hierarchical and complex designs, identification of the appropriate level for tests and full reporting of outcomes
[x]	[ ]	Estimates of effect sizes (e.g. Cohen's *d*, Pearson's *r*), indicating how they were calculated

Our web collection on statistics for biologists contains articles on many of the points above.

Software and code

Policy information about availability of computer code

Data collection	not applicable
Data analysis	not applicable

For manuscripts utilizing custom algorithms or software that are central to the research but not yet described in published literature, software must be made available to editors and reviewers. We strongly encourage code deposition in a community repository (e.g. GitHub). See the Nature Portfolio guidelines for submitting code & software for further information.

Data

Policy information about availability of data

All manuscripts must include a data availability statement. This statement should provide the following information, where applicable:

- Accession codes, unique identifiers, or web links for publicly available datasets
- A description of any restrictions on data availability
- For clinical datasets or third party data, please ensure that the statement adheres to our policy

All data generated or analysed during this study are included in this published article. All SOI ROV dive videos are open access at you tube (youtube.com/ @SchmidtOcean/videos).

Research involving human participants, their data, or biological material

Policy information about studies with human participants or human data. See also policy information about sex, gender (identity/presentation), and sexual orientation and race, ethnicity and racism.

Reporting on sex and gender	not applicable
Reporting on race, ethnicity, or other socially relevant groupings	not applicable
Population characteristics	not applicable
Recruitment	not applicable
Ethics oversight	not applicable

Note that full information on the approval of the study protocol must also be provided in the manuscript.

Field-specific reporting

Please select the one below that is the best fit for your research. If you are not sure, read the appropriate sections before making your selection.

☐ Life sciences ☐ Behavioural & social sciences ☒ Ecological, evolutionary & environmental sciences

For a reference copy of the document with all sections, see nature.com/documents/nr-reporting-summary-flat.pdf

Ecological, evolutionary & environmental sciences study design

All studies must disclose on these points even when the disclosure is negative.

Study description	To test the hypothesis of larval transit through the Earth's crust, we chose a vent site, which we named Fava Flow Suburbs, at the 9° 50'N EPR at 2515 m water depth.
Research sample	We selected small, discrete clumps of R. pachyptila and O. alvinae tubeworms (N = 1), a mix of tubeworms and Bathymodiolus thermophilus mussels (N = 3), or mussels (N = 2) along a hydrothermal flux gradient growing above cracks on lobate lava. While attempting an in situ experiment to demonstrate larval settlement from beneath the surface, we discovered animal life beneath the surface of the crust.
Sampling strategy	We used the ROV SuBastian to lift whole lobate lava shelves and discovered caves of ~10 cm height below the ~10-15 cm thick lava shelf which were inhabited by microbes and various animals.<br>A sample size of N = 6 was used, to allow for as much replication as possible given the limited available time by the remotely operated vehicle (ROV).
Data collection	From manuscript:<br>Methods<br>Collection of lobate lava: ROV SuBastian from Schmidt Ocean Institute (SOI) was used during expedition FKt230629 with R/V Falkor (too) and dives S0543-S0560 to explore subsurface life at the hydrothermal vent site Fava Flow Suburbs at 9°50'N EPR at 2515 m depth in July 2023 (Table 1). Six patches, all of a size of ~ 50 x 50 cm, and located within an area of ~ 20 by 20 meters were studied (Figure 1, Supplementary Figure 1). Because the rock was impossible to break into small pieces, we lifted the entire lobate lava shelf. Prior to lifting, a small ~ 2 cm wide hole was drilled with a small ~ 30 cm long chisel to gently widen an existing crack, allowing in situ temperature measurements and sampling of fluid from the subsurface using an intake handle (13 mm diameter, WHOI), connected to a miniature magnetic drive gear pump (Suofu; NP060; with 40W rotor-less brushless direct current motor) and flexible water tank system (CAN-SB Marine Plastics) mounted on the ROV (Supplementary Figure 2). Afterwards, the ~ 2 cm wide hole was enlarged to allow the usage of a suction sampler. Finally, a large 1.5-meter-long pry bar was used to enter the hole diagonally into the cavity for ~80 cm. The lava shelf then was gently lifted and flipped up-side down, opening the view into the subseafloor cavities (Supplementary Movie 1). The whole procedure was filmed, and the bottom and the ceiling of the cavity visually inspected in situ. Short underwater video clips of cavity openings were made with Open Shot Video Editor (OpenShot Studios, LLC, Rockwall, Texas, US). After inspection during ROV dives, parts of the lava shelf were recovered by the ROV arm, placed into a sealed box on the ROV platform, and brought on board of the ship R/V Falkor(too). The lava shelf height was measured with a ruler onboard the ship. Cave height was estimated in situ based on the distance of ROV lasers.<br>Measurements of abiotic water parameters: The ROV SuBastian temperature probe PT100 was used to measure in situ temperature of ambient seawater, vent fluid among animal clumps, and vent fluid in the subsurface cavities (through the ~ 2 – 3 cm wide hole made with the chisel and in about 20 cm below the seafloor surface). Salinity, pH, oxygen and sulfide concentrations were analyzed from water samples taken into the flexible water tank system. Onboard the ship the pump was reversed and water was filled into a glass bottle to analyze pH and salinity with a Multi 340i sensor (WTW, Germany), oxygen concentration together with temperature with the dipping probe DP-PSt3 connected to a Fibox 4 trace oxygen meter (PreSenS, Germany), and ΣH2S (sum of all forms of dissolved sulfide 1) with the LaMotte sulfide test kit and measured with a HACH Lange DR 1900 photometer calibrated with the UniSense sulfide standard.<br>Macrofaunal analyses: Underwater videos and framegrabs were analyzed for the presence of vent fauna and identified following

	Desbruyères et al. 2. Sizes of tubeworms and other animals were estimated based on ROV laser distance in situ, or measured onboard of the ship directly with a ruler, or taking pictures of animals to compare them with respective scales of an object micrometer with a Canon EOS 550D camera mounted to a BMS Stereo Trino Zoom microscope.
Timing and spatial scale	Timing included July 13/15/17/18/19/21 in 2023 (see Table 1). Spatial scale included the chosen vent site Fava Flow with the six discrete patches (see Table 1 for longitude, latitude and depth).
Data exclusions	not applicable
Reproducibility	A total of 6 caves were studied. 5 of the 6 caves contained visible fauna.
Randomization	Not relevant. We could not select a priori the caves, as these were not visible before lifting the lava shelf. We have chosen 6 patches according to the visible megafauna on the surface, either only tubeworms, only mussels, or a mix of both.
Blinding	Not relevant. We could not select a priori the caves, as these were not visible before lifting the lava shelf. We have chosen 6 patches according to the visible megafauna on the surface, either only tubeworms, only mussels, or a mix of both.

Did the study involve field work? ☒ Yes ☐ No

Field work, collection and transport

Field conditions	All field conditions are described in Table 1.
Location	All locations are described in Table 1.
Access & import/export	We have added this information to the manuscript (methods)<br>We confirm that our research complies with all relevant regulations for obtaining biological material, collected in areas beyond national jurisdiction, and imported to and exported from Panama and sent to Austria and the Netherlands (Import and export permit to and from Panama, Ministerio de Desarrollo agropecuario (215390 - 215391, 215392-215398)<br>and Ministerio de Ambiente (PA-05-ARB-114-2023; PA-05-ARB-131-2023); import permit from Panama to Austria, Federal Ministry of Social Affairs, Health, Care and Consumer Protection (2023-0.250.333); import permit from Panama to Netherlands, NVWA (NVWA-0166861)).
Disturbance	We lifted 6 small lava shelfs of a small size (~ 50 x 50 cm) to minimize the disturbance.

Reporting for specific materials, systems and methods

We require information from authors about some types of materials, experimental systems and methods used in many studies. Here, indicate whether each material, system or method listed is relevant to your study. If you are not sure if a list item applies to your research, read the appropriate section before selecting a response.

Materials & experimental systems

n/a	Involved in the study
☒	☐ Antibodies
☒	☐ Eukaryotic cell lines
☒	☐ Palaeontology and archaeology
☐	☒ Animals and other organisms
☒	☐ Clinical data
☒	☐ Dual use research of concern
☒	☐ Plants

Methods

n/a	Involved in the study
☒	☐ ChIP-seq
☒	☐ Flow cytometry
☒	☐ MRI-based neuroimaging

Animals and other research organisms

Policy information about studies involving animals; ARRIVE guidelines recommended for reporting animal research, and Sex and Gender in Research

Laboratory animals	Study did not involve laboratory animals
Wild animals	Wild animals were observed with video by the ROV. The recovered lava shelfs sometimes contained animals from the ceiling of the cave. A few animals were caught when the parts of the lava shelf were recovered by the ROV arm, placed into a sealed box on the ROV platform, and brought on board of the ship R/V Falkor(too). These invertebrate animals were killed.
Reporting on sex	Sex was not determined, with the exception of four recovered Riftia tubeworms (2 males, 2 females).

Field-collected samples	No laboratory work was performed. The animals were photographed and morphologically identified. Four Riftia tubeworms (see wild animals above) were identified for this study on board of the ship and sex identified.
Ethics oversight	No ethical approval was required. Study area in international waters (areas beyond national jurisdiction). Non endangered invertebrate species only.

Note that full information on the approval of the study protocol must also be provided in the manuscript.

Plants

Seed stocks	not applicable
Novel plant genotypes	not applicable
Authentication	not applicable
